# Assessing mosquito dynamics and dengue transmission in Foz do Iguaçu, Brazil through an enhanced temperature-dependent mathematical model

**DOI:** 10.1371/journal.pone.0330902

**Published:** 2025-09-08

**Authors:** Caio S. Rauh, Eduardo C. Araujo, Fabiana Ganem, Raquel M. Lana, André S. Leandro, Caroline A. Martins, Flávio C. Coelho, Cláudia T. Codeço, Leonardo S. Bastos, Suani T. R. Pinho

**Affiliations:** 1 Instituto de Física, Universidade Federal da Bahia (UFBA), Salvador, Bahia, Brazil; 2 Universidade Tecnológica Federal do Paraná (UTFPR), Curitiba, Paraná, Brazil; 3 Programa de Computação Científica (PROCC), Fundação Oswaldo Cruz, Rio de Janeiro, Rio de Janeiro, Brazil; 4 Fundação Getúlio Vargas, Applied Mathematics School, Rio de Janeiro, Rio de Janeiro, Brazil; 5 Barcelona Supercomputing Center (BSC), Barcelona, Catalonia, Spain; 6 Centro de Controle de Zoonoses, Secretaria Municipal de Saúde, Foz do Iguaçu, Paraná, Brazil; 7 Unidade de Vigilância em Zoonoses, Secretaria Municipal da Saúde de Foz do, Iguaçu, Foz do Iguaçu, Brazil; 8 Laboratório de Mosquitos Transmissores de Hematozoários, Instituto Oswaldo Cruz, Fiocruz, Rio de Janeiro, Brazil; 9 Instituto de Matemática Pura e Aplicada e Tecnologia (IMPA Tech), Rio de Janeiro, Brazil; Instituto Nacional de Salud Publica, MEXICO

## Abstract

Dengue fever remains a major public health concern, requiring continuous efforts to mitigate its impact. This study investigates the influence of key temperature-dependent parameters on dengue transmission dynamics in Foz do Iguaçu, a tri-border municipality in southern Brazil, using a mathematical model based on a system of ordinary differential equations. The fitted model aligns well with observed data. To track changes in dengue transmission over time and detect epidemic onset, we calculated the effective reproduction number. Additionally, we explored the potential effects of climate variability on dengue dynamics. Our findings highlight the importance of vector population dynamics, climate, and incidence, offering insights into dengue transmission in Foz do Iguaçu. This research provides a foundation for optimizing intervention strategies in other cities, improving outbreak prediction, and supporting public health efforts in dengue control.

## Introduction

Dengue is a viral vector-borne disease with a complex transmission dynamic and fast-growing burden, posing challenges for surveillance and control programs worldwide. The dengue virus, classified into four serotypes (DENV1-4) [1], is endemic in tropical and subtropical regions of the world and has its distribution expanding to higher altitudes and latitudes, due to the erosion of climate barriers [2].

The primary dengue vectors, *Aedes aegypti* and *Aedes albopictus* mosquitoes, are highly anthropophilic and well adapted to urban environments [3]. In Brazil, *Ae. aegypti* reinvaded the country after elimination campaigns in the mid-$20^{\text{th}}$ century and is now present in most municipalities [4]. These mosquitoes also transmit Zika and Chikungunya viruses, which have been circulating in Brazil since 2014 [5–7].

Dengue transmission dynamics are inherently linked to climatic factors, as they influence the ecology of its invertebrate vectors. Vector density, often measured as the average number of mosquitoes per person, depends on the availability of breeding sites and suitable climate conditions for survival and reproduction [8,9]. Additionally, a substantial body of literature explores the effects of climate on the mosquito life cycle and vector capacity. For example, temperature affects the transmission cycle of dengue by shortening the extrinsic incubation period [10]. The role of temperature on dengue dynamics [9,11–13] is well recognized, with many countries using seasonal climate data to build predictive models and issue dengue warnings [8,14–17].

Favorable conditions for vector reproduction and carrying capacity, unplanned urbanization generating highly occupied regions but with disproportionate infrastructure, human behaviors and variables such as built-up areas, vegetation index, road density, proximity to water and characteristics of establishments are cited as the main drivers of dengue spread and are the variables most used to model vector abundance and dengue risk [18]. A study conducted by [19,20] indicates that Brazilian microregions with a previous baseline level of dengue transmission and a high degree of urbanization presented higher dengue incidence in recent years. Furthermore, climatic conditions and anomalies have exacerbated dengue incidence rates, even in areas with historically low transmission. Regions at higher altitudes, previously considered less susceptible to the disease, are now becoming increasingly vulnerable to dengue and other arboviruses. This situation is further exacerbated by the effects of initial epidemic waves, the rapid Aedes vector proliferation, and the virus introduction into a predominantly susceptible population [19].

Mathematical models are essential tools for understanding arbovirus transmission dynamics. By incorporating both human and vector components and being parameterized with comprehensive datasets, these models provide valuable insights for testing hypotheses about disease transmission and exploring intervention scenarios. Such exploratory simulations can predict future outbreaks, improve preparedness, and support the planning of effective vector control interventions and timely responses – a critical challenge for public health authorities. However, despite this potential, modelers rarely have access to long-term measures of both entomological (adult mosquito indices) and epidemiological time series (incidence) to fit these models and test hypotheses [8,21,22]

Foz do Iguaçu, located in southern Brazil, serves as a unique and strategic site for assessing dengue transmission at the fringe of the disease distribution. Situated in the tri-border area with Argentina (Puerto Iguazu) and Paraguay (Ciudad del Este), it is a critical point for studying transboundary dengue dynamics. Furthermore, it is one of the most visited cities in Brazil, receiving millions of visitors every year. The region is characterized by a humid subtropical climate with cool winters and warm summers. Foz do Iguaçu has recorded dengue fever since 1998, with nine outbreaks between 2010 and 2021, according to [23] data.

Since 2017, the city has operated a unique integrated entomological and epidemiological surveillance system. The system monitors a large set of adult mosquito traps across the city every two months and detects viruses in mosquitoes and human samples, showing good predictive value [24]. This system detected a drastic increase in mosquito abundance during the 2020-2022 period. This rich dataset provides an opportunity to investigate the relationship between mosquito dynamics and dengue outbreaks using mathematical models. With this aim, in this paper, we propose an enhanced deterministic model assuming functions of temperature and time for the entomological parameters to analyze and simulate the temporal dynamics of dengue in Foz do Iguaçu, evaluating the influence of climate on the vector population and their consequent impact on dengue incidence.

## Materials and methods

### Data description and processing

Foz do Iguaçu is situated in the Western part of the Paraná State, Brazil, at 25°32’49” S latitude and 54°35’18” W longitude. The city stands at an elevation of 164 meters above sea level and covers a total area of 618.352 km^2^. The mean annual temperature in the region is 20.4°C, and the annual average rainfall amounts to 1,800 mm. The municipality is home to a population of 256 thousand inhabitants and has a high HDI (Human Development Index) of 0.751 [25].

#### Epidemiological data.

The anonymized line list of dengue cases by place of residence in Foz do Iguaçu was obtained from the Notifiable Diseases Information System (SINAN) [23], provided by the Foz do Iguaçu Health Department. The dataset contains daily georeferenced information on reported cases, for the period between 07/01/2010 and 30/12/2022. In this period, 23.52% of the notified cases were lab-confirmed, and 2.05% had serotype information.

#### Entomological data.

Data on mosquito traps are aggregated according to a systematic bimonthly collection. We use data (odd months) from 09/01/2017 to 13/05/2022, totaling 33 collections about trapped mosquitoes provided by the city’s Zoonosis Control Center (CCZ). There are 2500 georeferenced mosquito traps (Adultrap) distributed throughout the city. The traps are designed to attract and capture female mosquitoes seeking oviposition sites. The entomological data came from the Foz do Iguaçu citywide vector surveillance program that bimonthly deploys adult mosquito traps throughout the city.

Adultrap data and indices have recognized operational constraints and, to date, long-term datasets of this type remain scarce in Brazil. Our data represents an important but still local resource. Evaluations in Foz do Iguaçu show that these indices are more responsive to changes in mosquito density than traditional larval surveys [24]. Although coverage is not in 100% of the city, the trap network captured clear seasonal and spatial signals, which we confirmed in exploratory analyses, giving confidence in its use as a model input.

We calculated the MFAI (Mean Female *Aedes sp.* index) as the ratio between the number of females captured and the number of traps inspected[24]. Based on observations from Foz do Iguaçu field entomologists, mosquito counts per trap are considered reliable only for those captured within a maximum of 15 days before the collection date. Mosquitoes collected earlier than this period are at a significant risk of deterioration or predation. Consequently, for model fitting purposes, we assume that the MFAI metric represents the mosquitoes captured in the trap within the last 15 days.

#### Climate data.

Meteorological data were collected by the Meteorological Station in Foz do Iguaçu and obtained from the Paraná Meteorological System (SIMEPAR) (http://www.simepar.org/prognozweb/simepar). The dataset includes daily records of average, minimum, and maximum temperatures, precipitation, average humidity, and wind speed for the period from January 1, 2010, to December 30, 2022. Missing values in the meteorological data were imputed using the seven-day moving average.

### Mathematical modelling

#### Complete dengue model.

**Fig 1:**
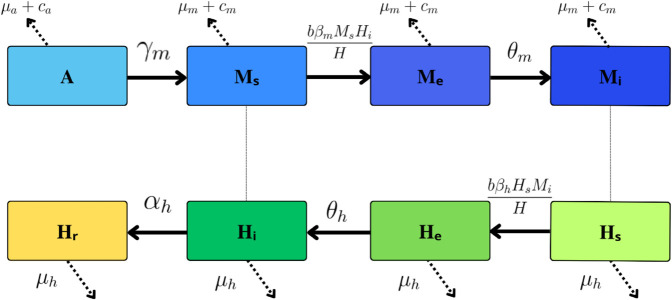
Complete model diagram. Schematic representation of the model proposed by [26], and further refined in this study. The diagram illustrates the population compartments and their transition rates, with arrows indicating the direction of population flow. Dashed lines highlight interactions between the mosquito and human populations.

Aiming to mathematically describe dengue transmission dynamics in Foz do Iguaçu, we enhanced a deterministic temperature-dependent model of transmission, previously developed by [26], referred here as *complete dengue model* (Fig 1, Eq 1). The model describes the transmission of a single dengue serotype between mosquitoes and humans in a homogeneous setting where *M* consists of the adult female mosquito population and *A* aquatic stage mosquitoes. The total number of adult females, *M*, is divided into three subdivisions: susceptible *M*_*s*_, exposed *M*_*e*_ and infected mosquitoes *M*_*i*_.

As for the total human population, it is considered constant and has four compartments: susceptible *H*_*s*_, exposed *H*_*e*_, infectious *H*_*i*_ and recovered *H*_*r*_. Thus, we have $M=M_{s}\;+\;M_{e}\;+\;M_{i}$ and $H=H_{s}\;+\;H_{e}\;+\;H_{i}\;+\;H_{r}$.

Fig 1 presents a diagram of the complete dengue model, highlighting both the temperature-dependent entomological parameters and those assumed to remain constant. These parameters include the oviposition rate δ(T(t)), the emergence rate of mosquitoes from the aquatic phase $\gamma_{m}(T(t))$, and the mortality rates for both the aquatic phase $\mu_{a}(T(t))$ and adult mosquitoes $\mu_{m}(T(t))$. The per capita mosquito biting rate, denoted as *b*(*T*(*t*)), represents the average number of bites per mosquito per day. The transmission probabilities from humans to mosquitoes and vice versa are given by $\beta_{m}(T(t))$ and $\beta_{h}(T(t))$, respectively. Additionally, $\theta_{m}(T(t))$ and $\theta_{h}$ represent the rates at which mosquitoes and humans become infectious. Their reciprocal values, $1/\theta_{m}(T(t))$ and $1/\theta_{h}$, correspond to the extrinsic and intrinsic incubation periods of the virus, respectively. The mosquito carrying capacity is denoted as *C*(*t*) and is initially assumed constant, though this assumption is later relaxed, as discussed in the results. This parameter represents the maximum number of aquatic-stage mosquitoes (eggs, larvae, and pupae) that the environment can support, reflecting primarily the availability of breeding sites such as artificial containers and natural water-holding structures but also influenced by climatic, ecological, and urban factors. The human death rate is represented by $\mu_{h}$, while *c*_*a*_ and *c*_*m*_ describe the death rates, induced by control efforts, of aquatic and adult mosquitoes, respectively.

We reproduce in (1), the system of nonlinear differential equations of the complete model proposed by [26].

$$\left\{ \begin{array}{c} \frac{dA}{dt}=k\delta (T(t))\left(1-\frac{A(t)}{C(t)}\right)M(t)-(\gamma_{m}(T(t))+\mu_{a}(T(t))+c_{a})A(t),\\ \frac{dM_{s}}{dt}=\gamma_{m}(T(t))A(t)-\frac{b(T(t))\beta_{m}(T(t))M_{s}(t)H_{i}}{H}-(\mu_{m}(T(t))+c_{m})M_{s}(t),\\ \frac{dM_{e}}{dt}=\frac{b(T(t))\beta_{m}(T(t))M_{s}(t)H_{i}(t)}{H}-(\theta_{m}(T(t))+\mu_{m}(T(t))+c_{m})M_{e}(t),\\ \frac{dM_{i}}{dt}=\theta_{m}(T(t))M_{e}(t)-(\mu_{m}(T(t))+c_{m})M_{i}(t),\\ \frac{dH_{s}}{dt}=\mu_{h}(H(t)-H_{s}(t))-\frac{b(T(t))\beta_{h}(T(t))H_{s}(t)M_{i}(t)}{H},\\ \frac{dH_{e}}{dt}=\frac{b(T(t))\beta_{h}(T(t))H_{s}(t)M_{i}(t)}{H}-(\theta_{h}+\mu_{h})H_{e}(t),\\ \frac{dH_{i}}{dt}=\theta_{h}H_{e}(t)-(\alpha_{h}+\mu_{h})H_{i}(t),\\ \frac{dH_{r}}{dt}=\alpha_{h}H_{i}(t)-\mu_{h}H_{r}(t). \end{array}\right.$$ (1)

To fit this model to Foz do Iguaçu’s data, it was necessary to modify some of its assumptions, as explained in subsection Temperature-dependent entomological parameters

#### Mosquito capture model.

We proposed a sub-model containing only the mosquito population, without infection to fit the mosquito component of the complete model with available trap data, refereed as *mosquito capture model*. In this model, we additionally represented the trapping process, in a way similar to [27]. The population of trapped mosquitoes is included in the model as an extra equation, however, the variables and parameters are the same as in the *complete model*.

$$\left\{ \begin{array}{c} \frac{dA}{dt}=k\delta (T(t))\left(1-\frac{A(t)}{C(t)}\right)M(t)-(\gamma_{m}(T(t))+\mu_{a}(T(t)))A(t),\\ \frac{dM}{dt}=\gamma_{m}(T(t))A(t)-\mu_{m}(T(t))M(t)-T_{rapped}(t),\\ \frac{dT_{rapped}}{dt}=\alpha \frac{N_{tr}}{H_{o}}M(t). \end{array}\right.$$ (2)

Note that the *mosquito capture model* is a particular case of the *complete model* for which there is no infection; due to that, there are only the aquatic and adult phases of the mosquito population. The third equation counts the captured mosquitoes, where the variable *T*_*rapped*_(*t*) represents the accumulated number of mosquitoes captured during the surveillance period from time 0 until t.

To fit the model with data from mosquitoes captured in each bimonthly collection, we calculate the MFAI as in [27], which is the ratio between the number of females captured and the number of traps inspected in the collection. The theoretical MFAI is calculated every two months *t*_*b*_ by the equation,

$$MFAI_{teo}(t_{b})=\frac{Trapped(t_{b})-Trapped(t_{b}-1)}{N_{tr}}.$$ (3)

#### Temperature-dependent entomological parameters.

Following [28], we assume that the entomological parameters show temporal variation associated with the daily temperature in Foz do Iguaçu. Table 1 describes the complete model parameters including the entomological ones, as well as the range of values.

**Table 1:** Complete dengue model: symbols, description, values, and references.

Parameter	Biological meaning	Range of values	Value/Comment	Sources
*N*	Foz do Iguaçu’s population	–	256088	[29]
*N*_*tr*_	number of mosquito traps	–	2412	data*
*H*_*o*_	number of households	–	102751	data*
*α*	mosquito trap capture rate	0–1	0.02	data*
$\mu_{h}$	human mortality rate	$[3.425\;-\;5.479]\;\times \;10^{-5}$day^−1^	$3.605\cdot 10^{-5}$	[26]
$\theta_{h}$	intrinsic incubation rate	0.1–0.33 day^−1^	0.125	[30]
$\alpha_{h}$	recovering rate	0.083–0.333 day^−1^	0.125	[31]
*k*	fraction of female hatched from all eggs	0–1	0.5	[26]
*c*_*a*_	aquatic control effort rate	0–1	0	[26]
*c*_*m*_	adult control effort rate	0–1	0	[26]
δ(T(t))	oviposition rate	0–8.8960 day^−1^	polynomial fitted	[32]
$\mu_{m}(T(t))$	mosquito mortality rate	0.0301–1 day^−1^	polynomial fitted	[32]
$\mu_{a}(T(t))$	aquatic mortality rate	0.0234–1 day^−1^	polynomial fitted	[32]
$\gamma_{m}(T(t))$	aquatic transition rate	0–0.1724 day^−1^	polynomial fitted	[32]
$\theta_{m}(T(t))$	extrinsic incubation rate	0.02–0.2 day^−1^	according to Eq (4)	[32]
*b*(*T*(*t*))	bite per mosquito per day	0–1 day^−1^	according to Eq (5)	[33]
$\beta_{m}(T(t))$, $\beta_{h}(T(t))$	effective contact rates	0–1	according to Eq (5)	[33]
*a*_*b*_	coefficient of the biting rate function *b*(*t*)	0–1	$5.22\;\times \;10^{-4}$	fitted
*a*_*m*_	coefficient of effective contact rate function $\beta_{m}(t)$	0–1	$4.203\;\times \;10^{-4}$	fitted
*a*_*h*_	coefficient of effective contact rate function $\beta_{h}(t)$	0–1	$1.05\;\times \;10^{-3}$	fitted
*C*(*t*)	mosquitoes carrying capacity	$[0-3]\times 10^{5}$	according to Eq (6)	fitted
*C*_0_	initial carrying capacity	0–3	1.33	fitted
*b*_*cap*_	coefficient of carrying capacity function	0–1.2	0.3165	fitted
𝜖	carrying capacity threshold	0–1820 days	909	fitted
*ϕ*	proportion of susceptible individuals	0–1	0.14	fitted

* data provided by Foz do Iguaçu Health Department and Foz do Iguaçu Zoonosis Control Center (CCZ).

We used the values for five entomological parameters that vary with temperature, δ(T(t)), $\mu_{m}(T(t))$, $\mu_{a}(T(t))$, $\gamma_{m}(T(t))$ and $\theta_{m}(T(t))$ proposed by [32]. The expressions for parameters *δ*, $\mu_{m}$, $\mu_{a}$, $\gamma_{m}$ are obtained using the methodology in [28,32] fitting entomological data to a polynomial of degree n, $P_{n}(T)=b_{0}+b_{1}T+\ldots +b_{n}T^{n}$. Now, for the extrinsic incubation $\theta_{m}(T(t))$, according to [34], it depends on the temperature as follows:

$$\theta_{m}(T(t))=\frac{T(t)-T_{m}}{T_{s}},$$ (4)

where *T*(*t*) is the average daily temperature in degrees Celsius °C, *T*_*s*_ is the thermal sum (°C × day), measured in degree-days representing the accumulation of temperature units over time, and *T*_*m*_ is the threshold of temperature below which dengue virus cannot multiply, hence $T\;>\;T_{m}$. We assume $T_{m}=14^{\circ }C$ and $T_{s}=135^{\circ }C\;\times \;day$ according to [12]. Note that the values of this function choice align with the extrinsic incubation datasets analyzed in [30].

Using the above expressions, and the daily temperature of Foz do Iguaçu as input, we calculated the daily varying entomological parameters for the model.

Between 2010 and 2022, the minimum temperature in Foz do Iguaçu was $T_{min}=-1.8^{\circ }C$ and the maximum $T_{max}=41.8^{\circ }C$. Parameters extrapolations were proceeded based on biological literature regarding mosquito survival outside these ranges from [28]. Different temperature intervals are defined for the interpolation of each parameter. Except for $\theta_{m}$, which, due to its formulation, does not allow values of $T\leq 14^{\circ }C$. Parameter values for temperatures outside the interpolation range were fixed at their extreme values within the range as follows.

It is important to address the borders from the entomological parameters ranges of variation with temperature. For the oviposition rate, from [28], there is not any oviposition for the following temperatures: $\delta (T\leq 10.8^{\circ }C)=\delta (T\geq 37.3^{\circ }C)=0$.

Concerning the aquatic transition rate $\gamma_{m}$, for the following temperature ranges, there is no aquatic transition because the aquatic mortality rate is high and the oviposition rate is almost zero so the fitted polynomial crosses the temperature axis on: $\gamma_{m}(T\leq 12.0^{\circ }C)=\gamma_{m}(T\geq 40.3^{\circ }C)=0$. For the average aquatic mortality rate $\mu_{a}$, from data on [28], larva mortality is very high for low and high temperatures, so: $\mu_{a}(T\leq 5.2^{\circ }C)=1.0$. For the average adult mortality rate $\mu_{m}$, we see from the data on [28] that for very low temperatures, the mortality $\mu_{m}(T\leq -0.9^{\circ }C)=1.0$. For $b,\beta_{m},\beta_{h}$ is $b,\beta_{m},\beta_{h}(T\leq 18.0^{\circ }C)=b,\beta_{m},\beta_{h}(T\geq 34.0^{\circ }C)=0$. The extrinsic incubation rate $\theta_{m}$ is defined as $\theta_{m}(T\leq 16.4^{\circ }C)=0.02$ e $\theta_{m}(T\geq 38^{\circ }C)=0.2$.

The parameters *b*(*T*(*t*)), $\beta_{m}(T(t))$ and $\beta_{h}(T(t))$ are described by the asymmetric Brière function of temperature *T*(*t*) [11], which depends on time *t*.

$$𝔅(T(t))=aT(t)(T(t)-T_{min})(T_{max}-T{)}^{1/2}.$$ (5)

**Fig 2:**
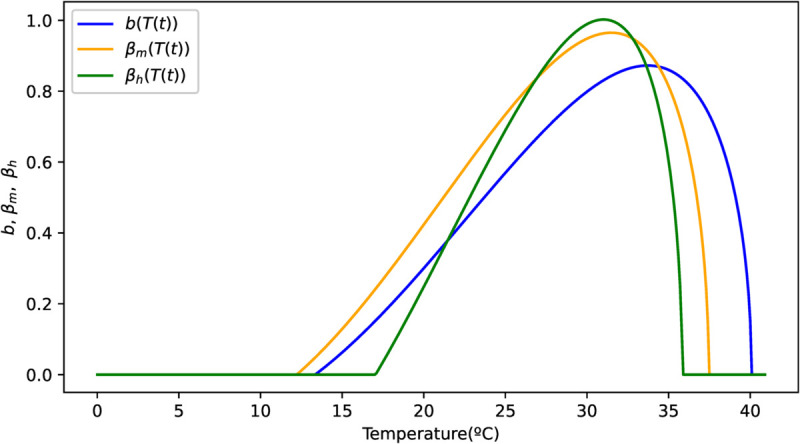
Functional form of parameters *b*(*T*(*t*)), $\beta_{m}(T(t))$, and $\beta_{h}(T(t))$. The temperature dependence of these parameters is modeled using the Brière function (Eq 5), fitted to dengue case data. The fitted coefficients are: $a_{b}=5.0366\cdot 10^{-4}$, $a_{m}=6.5093\cdot 10^{-4}$, and $a_{h}=1.0546\cdot 10^{-3}$.

Following [11], we set for *b*(*T*(*t*)) the following temperatures: $T_{min}=13.35^{\circ }C$ and $T_{max}=40.08^{\circ }C$. Now for $\beta_{m}(T(t))$ we have $T_{min}=12.22^{\circ }C$ and $T_{max}=37.46^{\circ }C$ and for $\beta_{h}(T(t))$, $T_{min}=17.05^{\circ }C$ and $T_{max}=35.83^{\circ }C$. Assuming that virus transmission occurs only within this temperature range. In cases where 𝔅(T(t))<0, this value is replaced by zero. The empirical coefficient *a* is fitted in the complete model for the three parameters *b*(*T*(*t*)), $\beta_{m}(T(t))$, and $\beta_{h}(T(t))$ (see subsection Sensitivity analysis and model calibration). Fig 2 shows the behavior of the three parameters described by the Brière function.

#### Carrying capacity.

**Fig 3:**
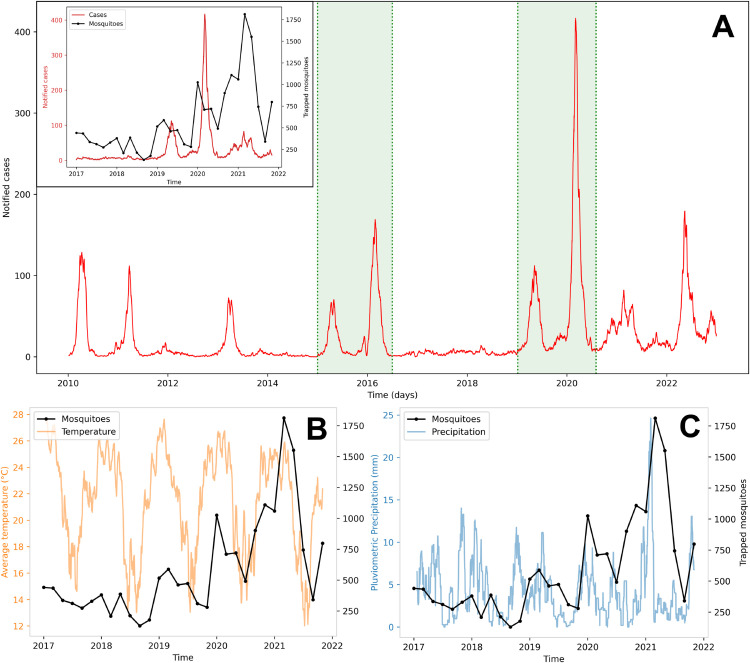
Foz do Iguaçu datasets. Panel showing the datasets used in this study: a) Time series of dengue cases in Foz do Iguacu, Brazil, from 2010 to 2022. The shaded green area highlights the periods used for curve fitting. The inset compares the number of trapped mosquitoes and dengue cases from 2017 to 2022. (b) and (c) Time series of bimonthly trapped mosquitoes alongside temperature and precipitation, respectively. Both are smoothed with moving averages (two-week for temperature and three-week for precipitation) over the period with available mosquito data.

Thus, for the carrying capacity parameter, a significant increase in captured mosquitoes is observed from 2019 onwards during the 2017–2021 period (Fig 3). This rise does not appear to be justified by significant variations in temperature or precipitation. Additionally, the number of traps analyzed remained nearly constant throughout the study period. Other environmental factors may also contribute to the increase in carrying capacity, such as changes in human behavior during the COVID-19 pandemic, which reduced tourism and increased remote work activities.

To achieve the formulation of the carrying capacity, different models were tested, like the one proposed by [32], considering ranges of temperature and precipitation. Also, we consider a linear relationship with precipitation according to [35]. The best formulation was the one which provided the best goodness-of-fit with the captured mosquito data following a temporal dependence without explicit dependence with climate variables, the Heaviside function

$$C(t)=C_{0}+b_{cap}u[t-\epsilon ](t-\epsilon ).$$ (6)

In this expression, *C*_0_ represents the initial carrying capacity, *u*(*t*) is the Heaviside function, *b*_*cap*_ is a constant rate associated with the growth of carrying capacity, and 𝜖 is a constant parameter that represents the time at which the carrying capacity begins to increase. *C*(*t*) assumes values of the order of 10^4^.

Based on the above considerations about the entomological parameters and on the literature about other parameters, we complete Table 1 with the values range of the *complete model*, including the ones presented by the temperature-dependent functions of entomological parameters.

#### Mosquito capture model parameters.

For the mosquito model, we assume the trapping process described by $\alpha N_{tr}/H_{o}$, where *α* is the trap attractiveness (attracted mosquito proportion by the trap within a household), and *N*_*tr*_ and *H*_*o*_ are the number of traps and households in Foz do Iguaçu, respectively. Therefore, the ratio $N_{tr}/H_{o}$ is the trap density per household. We assume α=0.02 arbitrarily, meaning that 2% of mosquitoes in a trap are captured per day. This value is chosen as previous studies [36] indicate a low capture rate.

### Sensitivity analysis and model calibration

A sensitivity analysis using the Sobol’ method [37] was performed to identify which parameters should be fitted. In the mosquito trapping model, we examined the impact of parameters *δ*, $\gamma_{m}$, $\mu_{a}$,$\mu_{m}$, *C*_0_, 𝜖 and *b*_*cap*_ on the total number of trapped mosquitoes (2). In the complete dengue model (1), we assessed the influence of parameters *a*_*b*_, $a_{\beta_{m}}$, $a_{\beta_{h}}$, *C*_0_, $\alpha_{h}$, and $\theta_{h}$ on the total number of infected humans.

After analyzing its sensitivity, the parameters *C*_0_, *b*_*cap*_, 𝜖, *ϕ*, *a*_*b*_, $a_{\beta_{m}}$, $a_{\beta_{h}}$, *a*_*h*_ were fitted in the models. To achieve this, we use the *lmfit* package of Python, based on the *Levenberg-Marquardt* (damped least-squares) fitting method [38].

For the mosquito model calibration, we use the trap data to estimate the coefficients *C*_0_, *b*_*cap*_ and 𝜖 of the carrying capacity equation (6).

With the fitted mosquito model, we proceed to fit the parameters $\phi ,a_{b},a_{m},a_{h},C_{0}$, of the complete model, using the time series of cases, where *ϕ* refers to the proportion of susceptible individuals and *a*_*b*_, *a*_*m*_, *a*_*h*_ correspond to the parameter *a* in the respective Brière functions (Eq 5) for *b*(*T*(*t*)), $\beta_{m}(T(t))$, and $\beta_{h}(T(t))$ and *C*_0_ the initial carrying capacity.

When we fit the model to the data, the following expression is minimized:

$$\frac{1}{N}\sum_{i=1}^{N}(\hat{Y}(t_{i};\phi ,a_{b},a_{m},a_{h},C_{0})-Y(t_{i}){)}^{2}.$$ (7)

Here, $\hat{Y}(t_{i})$ represents the new infections ($\theta_{h}H_{e}(t_{i})$) from the complete model, and *Y*(*t*_*i*_) represents the daily reported new cases data in the same period.

### Reproduction number of dengue in Foz do Iguaçu

The basic reproduction number *R*_0_ is a fundamental epidemiological parameter defined as the average number of secondary infections caused by a single infectious individual when introduced into a completely susceptible population. It is a critical threshold for epidemic invasion: if $R_{0}\;>\;1$ the epidemic grows, whereas if $R_{0}\;<\;1$ the transmission is not self-sustaining, and the outbreak will subside.

Here, we use the expression for the dengue basic reproduction number, as derived from the complete model [26] using the next-generation matrix method. This calculation assumes an exponential increase in cases at the beginning of an epidemic. The expression for *R*0 is presented in Eq 8:

$$R_{0}=\sqrt{\left(\frac{\Lambda }{\theta_{m}(T(0))+\mu_{m}(T(0))}+1\right)\left(\frac{\Lambda }{\theta_{h}+\mu_{h}}+1\right)\left(\frac{\Lambda }{\mu_{m}(T(0))}+1\right)\left(\frac{\Lambda }{\alpha_{h}+\mu_{h}}+1\right)},$$ (8)

where the force of infection Λ corresponds to the exponent of new cases at the beginning of the epidemics.

To calculate *R*_0_ from data, we need to define the time interval for which there is exponential growth. For that time interval, it results in a linear growth in the plot of new cases against the cumulative number of cases.

Following the initial surge, the temporal progression of the epidemic no longer remains constant. Its characterization is now quantified by the time-dependent reproduction number, *R*(*t*). An expression for *R*(*t*) was derived from the complete model by [26] using the method described in [39]. Thus, *R*(*t*) is estimated by:

$$R(t)=\sqrt{\frac{b(t)}{\int_{a=0}^{\infty }b(t-a)g(a)\;da}},$$ (9)

where *b*(*t*) is the number of new cases at week *t* and *g*(*a*) is the dengue generation time interval distribution, which is defined as the probability distribution of the time an infected individual takes to infect a secondary case. In [26], following [40], the expression is

$$g(t)=\sum_{i=1}^{4}\frac{s_{1}(t)s_{2}(t)s_{3}s_{4}e^{-s_{i}(t)t}}{\prod_{j=1,j\neq i}^{4}(s_{j}(t)-s_{i}(t))},$$ (10)

where $s_{1}(t)=\theta_{m}(T(t))+\mu_{m}(T(t))+c_{m}(t)$, $s_{2}(t)=\mu_{m}(T(t))+c_{m}(t)$, $s_{3}=\theta_{h}+\mu_{h}$, $s_{4}=\alpha_{h}+\mu_{h}$. The parameters and their values are presented in Table 1.

Unlike [26], which utilized fixed temperature values for the entomological parameters, our analysis for Foz do Iguaçu considers the parameters $\theta_{m}(T(t))$ and $\mu_{m}(T(t))$ as functions of the observed temperature data.

## Results

The results will be presented in four subsections. First, we show time series data for dengue cases and trapped mosquitoes with temperature and precipitation. Then, we present the mosquito dynamics model in section Mosquito dynamics, followed by the dengue dynamics in section Dengue dynamics and, at last, the reproduction number in section Basic and Effective reproduction numbers for epidemic years.

### Dengue cases and trapped mosquitoes

The time series data from mosquitoes and dengue cases in Foz do Iguaçu are illustrated in Fig 3. Fig 3b and c specifically show the time series for trapped mosquitoes, along with temperature and precipitation, respectively.

**Fig 4:**
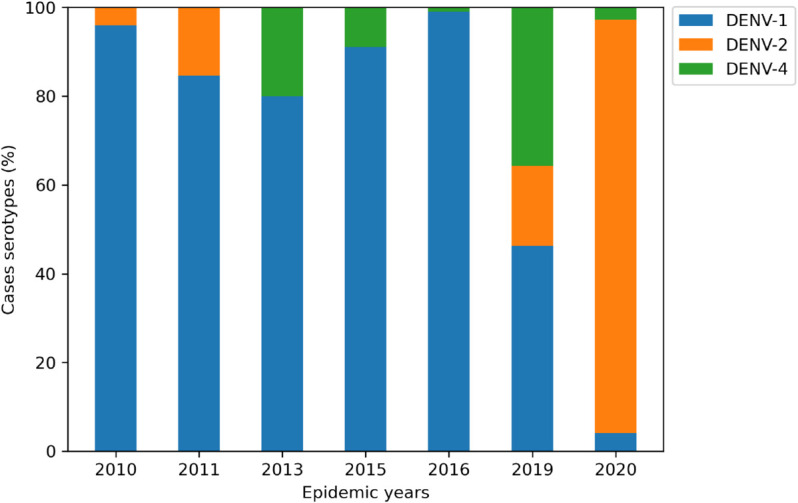
Serotype data for epidemic years. Description of the predominant DENV serotypes between 2010 and 2020 in Foz do Iguaçu, Brazil. It shows that DENV-1 was prevalent between 2010 and 2016 while we observed a strong detection of DENV-2 in 2020.

From 2010 to 2021, Foz do Iguaçu experienced nine dengue outbreaks, in the years 2010, 2011, 2013, 2015, 2016, 2019, 2020, and 2021. For model fitting, we selected the 2015-2016 and 2019-2020 outbreaks (highlighted by green shaded areas in Fig 3 because these epidemic years exhibited double peaks, allowing us to test the model’s ability to reproduce complex transmission patterns. Notably, the 2015–2016 epidemic was characterized by the near exclusive circulation of DENV-1, while the 2019–2020 period involved co-circulation of DENV-1, DENV-2, and DENV-4 in 2019, followed by DENV-2 predominance in 2020 (Fig 4). While our model assumes the transmission of a single serotype, we aimed to assess its capability to capture qualitative epidemic trends under different virological contexts. We also note that double-peak patterns may arise from multiple interacting mechanisms, including intra-seasonal variations in climatic factors like temperature and rainfall, the chaotic interplay between seasonal forcings, and the competition dynamics among co-circulating serotypes, not necessarily linked to serotype dynamics alone [41,42].

Furthermore, the occurrence of different DENV serotypes over the years is shown in 4.

### Mosquito dynamics

**Fig 5:**
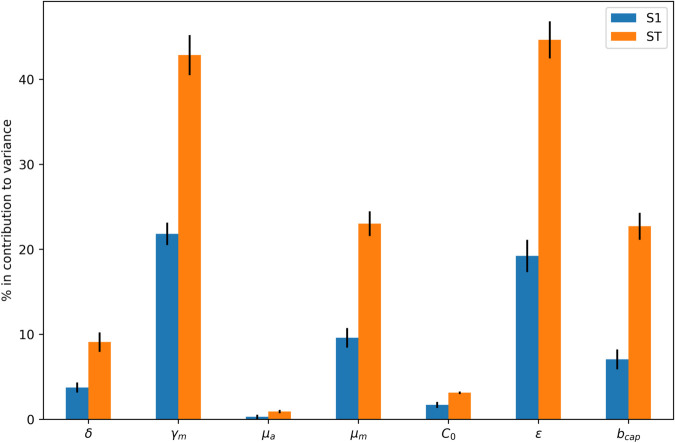
Mosquito model sensitivity analysis. Sobol’ sensitivity analysis evaluating the importance of selected parameters in the mosquito populational model, concerning the total number of trapped mosquitoes simulated. Here S1 and ST represent first-order and total sensitivity indices, respectively. The analyzed parameters and their corresponding scanned intervals were: *δ*: [0,9]day^−1^, $\gamma_{m}$: [0, 0.2]day^−1^, $\mu_{a}$: [0.0234,0.5]day^−1^, $\mu_{m}$: [0.0301, 0.109]day^−1^, *C*_0_: [10, 100], 𝜖: [0, 1000], *b*_*cap*_: [0.001, 1.2].

First, we performed a local sensitivity analysis where each parameter was varied while all the others were fixed. Then, a Sobol analysis was performed and the output is in Fig 5.

The parameters that affects the number of trapped mosquitoes the most were the carrying capacity threshold (𝜖) and the aquatic transition rate ($\gamma_{m}$).

#### Fitting and numerical simulations.

For the initial conditions of the mosquito model, we assumed M(0)=0.7N where *N* = 256088 is the total population of Foz do Iguaçu. A similar mosquito-to-human ratio was the same used by [43], based on measures from [44]. As for the aquatic phase, it was assumed *A*(0) = 0.85*C*_0_, with *C*_0_ being the carrying capacity at *t* = 0, as estimated by the model. The initial number of trapped mosquitoes was considered *T*_*rapped*_(0) = 0.

**Fig 6:**
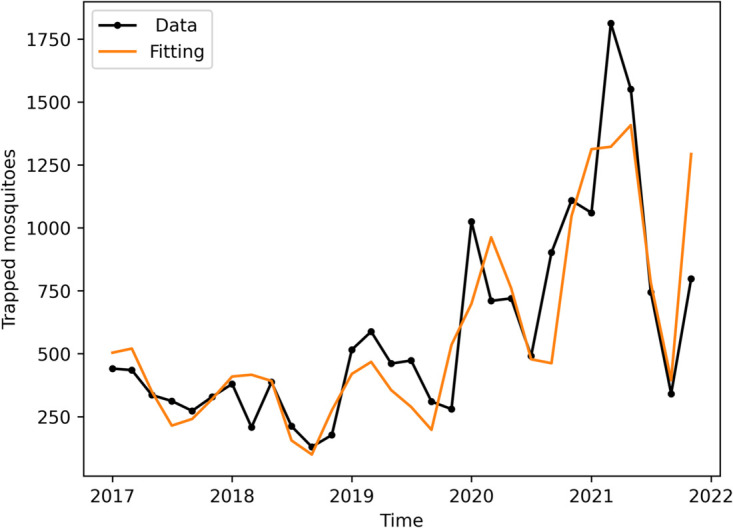
Mosquito populational model fitting. Bimonthly data of trapped mosquitoes and simulated curve of the mosquito model with the following fitted parameter values: $C_{0}=1.27\cdot 10^{5},b_{cap}=0.3165,\epsilon =909$ days.

In order to fit the parameters $C_{0},b_{cap}$ and 𝜖 of the carrying capacity *C*(*t*) (Eq 6) we use the captured mosquito data. The model fits the data well, predicting its seasonality and growth trend (Fig 6).

### Dengue dynamics

Here we consider the complete dengue model (1) based on the simulated results of the mosquito model, performing a sensitivity analysis and fitting the model with notified dengue cases.

**Fig 7:**
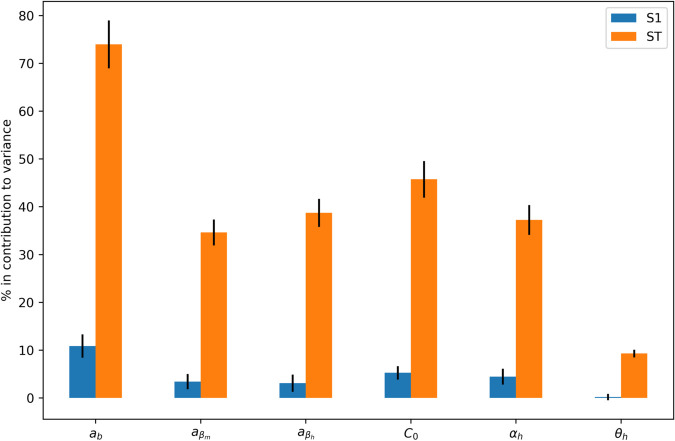
Complete model sensitivity analysis. Sobol’ sensitivity analysis evaluating the importance of selected parameters in the dengue transmission model, concerning the total number of infected humans simulated. Here S1 and ST represent first order and total sensitivity indices, respectively. The analyzed parameters and their corresponding scanned intervals were: *a*_*b*_:[0.0007,0.00138], $a_{\beta_{m}}:[0.0007,0.00138]$, $a_{\beta_{h}}:[0.0007,0.00138]$, *C*_0_:[12, 28], $\alpha_{h}:[0.083,0.25]day^{-1}$, $\theta_{h}:[1/12,1/3]day^{-1}$.

Fig 5 and 7 display the contributions of these parameters to the model, where S1 and ST represent the first-order, and total sensitivity indices, respectively. These indices provide a decomposition of output variance relative to each parameter. Notably, if the total-order indices (ST) are significantly larger than the first-order indices (S1), it indicates the presence of higher-order interactions among the parameters.

When integrating the complete model, the total number of simulated infected humans is used as input to assess the model’s sensitivity to each parameter. The results of this sensitivity analysis are shown in Fig 7. *a*_*b*_ associated with the bite rate *b* is the most significant parameter for the total number of infected humans.

#### Fitting and numerical simulations.

To fit the model to the dengue data during the 2015-2016 outbreaks, we used data from 01/01/2015 to 01/07/2016. The parameters $\phi ,a_{b}$, $a_{m},a_{h}$ and *C*_0_ were fitted. During both seasons considered here, we did not have mosquito population data to estimate C(t) so we assume that the carrying capacity was constant following the expression *C* = 0.7*NC*_0_. The initial conditions were: *A*(0) $=0.85C(0)=1.85\;\times \;10^{5}$, *M*_*s*_(0) = 0.7*N*, $M_{e}(0)=M_{i}(0)=0.7H_{i}(0)=13$, *H*_*s*_(0) = *N*− $H_{e}(0)-H_{i}(0)$ −*H*_*r*_(0) = 19243, $H_{e}(0)=18=H_{i}(0)$, $H_{r}(0)=(1-\phi )N=236881$.

As for the 2019-2020 seasons, we used data from 06/01/2019 to 30/12/2020. The model was fitted calibrating $\phi ,a_{b}$, $a_{m},a_{h},C_{0}$. For these outbreaks, the initial conditions for the mosquito compartment are obtained from the fitted mosquito model: $A(0)=1.33\;\times \;10^{5}$, $M_{s}(0)=3.63\;\times \;10^{5}$. For the human compartment, we set $M_{e}(0)=M_{i}(0)=$ 0.7*H*_*i*_(0) = 43, $H_{s}(0)=N\;-\;H_{e}(0)-H_{i}(0)-H_{r}(0)=31433$, *H*_*e*_(0) =  *H*_*i*_(0) = 62, $H_{r}(0)=(1-\phi )N$  = 226979. In Table 1, the range of values of the model parameters and the goodness-of-fit parameters estimated in the complete model for 2019-2020 are presented.

The dengue model captured well the seasonality of dengue in both periods. There was an overshoot in the 2016 outbreak, but all the other three were correctly described by the model.

### Basic and effective reproduction numbers for epidemic years

The estimated values of *R*_0_ and Λ for the epidemic years in Foz do Iguaçu are shown in Table 2. The precision of the *R*_0_ estimation is better during epidemic years where there is a well-defined exponential growth. The largest estimated values were found for the years 2010 and 2020. Fig 9 shows the linear phase for these years.

**Fig 8:**
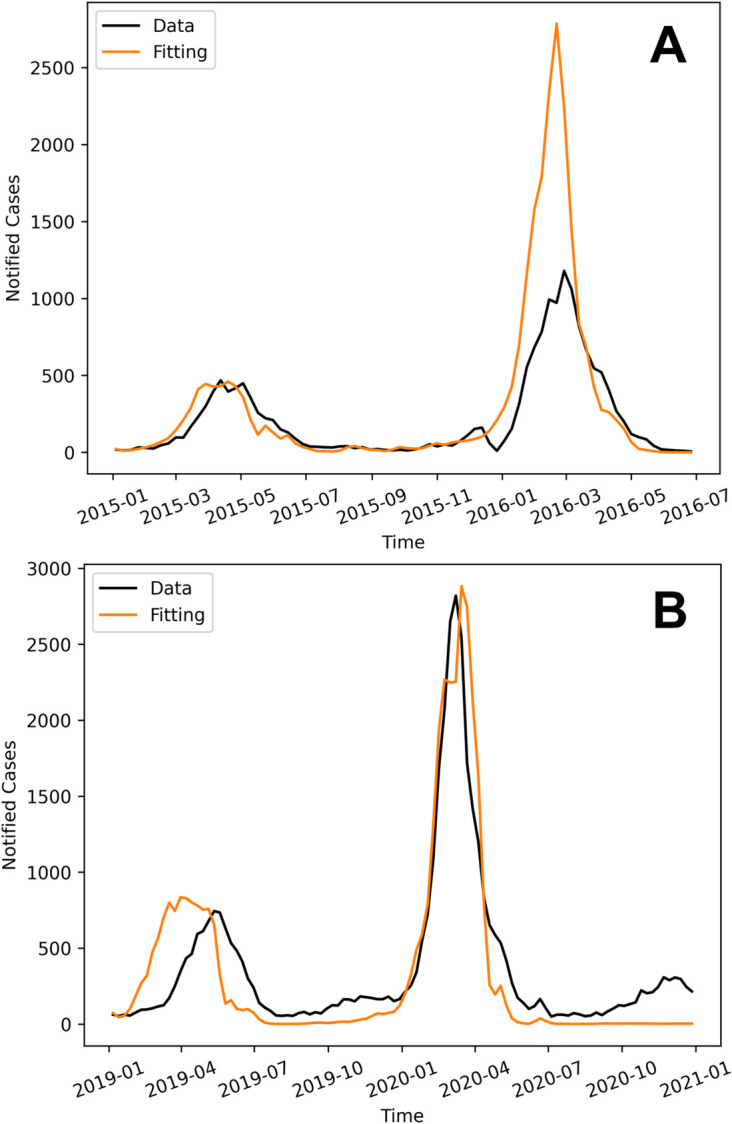
Dengue transmission model fittings. Comparison between observed and simulated weekly dengue cases. The black line represents notified dengue cases, while the orange line shows the simulated cases from the dengue transmission model with the following fitted parameter values for the outbreaks of 2015-2016 (a): ϕ=0.075, $a_{b}=5.316\;\times \;10^{-4}$, $a_{m}=6.616\;\times \;10^{-4}$, $a_{h}=1.06\;\times \;10^{-3}$, with constant carrying capacity *C*_0_ = 0.999. For the outbreak of 2019-2020 (b), we have: ϕ=0.14, $a_{b}=5.22\;\times \;10^{-4}$, $a_{m}=4.203\;\times \;10^{-4}$, $a_{h}=1.05\;\times \;10^{-3}$, with constant carrying capacity *C*_0_ = 1.33.

**Table 2:** Force of infection and basic reproduction number by epidemic year in Foz do Iguaçu, Brazil.

Year	Λ	$R_{0}$
**2010**	**0.61**	**4.22**
2011	0.31	2.37
2013	0.33	2.51
2015	0.33	2.47
2016	0.39	2.80
2019	0.25	2.10
**2020**	**0.48**	**3.33**
2021	0.32	2.46

*The two periods highlighted coincide with the entrance of serotypes DENV-1 and DENV-2, respectively.*

**Fig 9:**
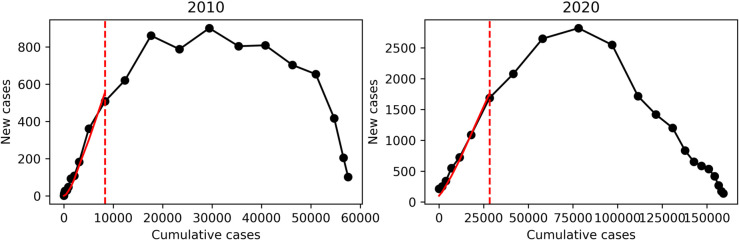
Basic reproductive number estimation through data. Graphical representation of the *R*_0_ estimation method based on dengue cases data. The black lines show the number of new cases plotted against the cumulative number of cases. The red dashed lines indicate the end of the linear phase, marking the point where the initial exponential growth approximation is no longer valid. The red solid lines represent the fitted curve used to estimate *R*_0_. Panel (a) corresponds to data from the 2010 dengue epidemic, while panel (b) shows data from 2020.

In Fig 9, we show the force of infection and basic reproduction number for the 2010 and 2020 epidemic years in Foz do Iguaçu. The two periods highlighted in bold in Table 2 coincide with the entrance of serotypes DENV-1 and DENV-2, respectively, where we can see the initial exponential growth as fitted by a straight line, using data and Eq (8).

**Fig 10:**
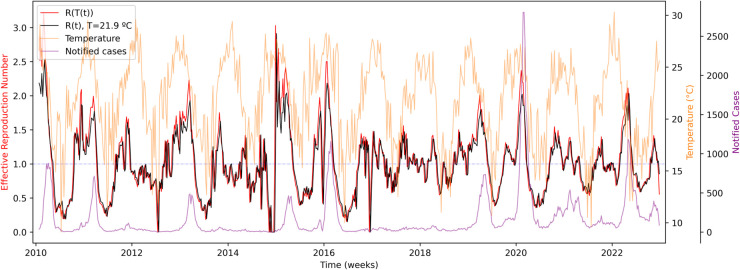
Effective reproduction number and temperature influence on dengue transmission. Time series showing the relationship between the effective reproduction number *R*(*t*), temperature, and reported dengue cases. The red line represents *R*(*T*(*t*)) varying dynamically with temperature, while the black line shows $R(T=21.9^{\circ }C)$ calculated with parameters in the mean daily temperature for Foz do Iguaçu. The orange line corresponds to the temperature time series, and the purple line represents the notified dengue cases data. Each parameter used is present in Table 1 and is multiplied by seven for weekly values.

To calculate the time-dependent reproduction number *R*(*t*), we consider the following dependence with temperature for parameters $\theta_{m}(T(t))$, $\mu_{m}(T(t))$ which takes part in the generation interval *g*(*t*) composing the rates of leaving the exposed and infectious compartments *s*_1_(*t*) and *s*_2_(*t*). The result of the calculated effective reproduction number can be seen in Fig 10, where we observe the instantaneous effect of temperature, in comparison to its value for an average temperature. The results suggest that high heat waves may promote large time intervals for which *R*(*t*) > 1. Also, note its high sensitivity to small variations of the weekly new cases and a tendency to grow near the outbreaks, as expected of a reproduction number, presenting the maximum *R*(*t*) values associated with 2020 and 2022 outbreaks.

## Discussion

In this work, we analyze the expansion of dengue in Foz do Iguaçu through a mathematical modeling perspective, counting on a rich dataset of mosquito and human infection data. This city has experienced a sequence of dengue outbreaks in the last two decades, as seen in figures 3a and 3b. In response, Foz do Iguaçu has implemented a comprehensive entomological surveillance program since 2017 [45], integrated with a sensible and responsive disease surveillance program.

Firstly, we aim to highlight the importance of collecting mosquito data to generate time series of mosquito populations, emphasizing its relevance to public health authorities. Second, we present a case study on dengue epidemics in an urban center with temperate climate, providing insights into disease dynamics under such conditions. Finally, the key contribution of this study is to underscore the significance of analyzing environmental influences on dengue dynamics from a modeling perspective. By incorporating the mosquito population dynamics, we can enhance the accuracy and depth of transmission models, leading to a more comprehensive understanding of dengue spread and its underlying drivers.

During the study period, the largest epidemics occurred in 2016 and 2020, both preceded by a shorter epidemic in the previous year. Sequences of epidemics like this suggest that transmission was interrupted during the winter to return in the next year. A pattern of two subsequent waves has been observed in other cities, like Taiwan [46], and Rio de Janeiro [47,48], and has been attributed to multiple serotypes or viral evolution.

Our analyses indicate that a one-serotype model with temperature as external forcing can produce the two-wave pattern observed in the data, with the second peak larger than the first. That is, the effect of temperature on mosquito dynamics in Foz do Iguaçu is sufficient to induce the observed dengue seasonality.

We note that 2010 and 2020 were each dominated by a single dengue serotype, justifying our one-strain approach for those years. However, the 2019 outbreak involved substantial co-circulation of DENV-1, DENV-2, and DENV-4, which our current model does not capture. Multi-strain frameworks can reproduce complex epidemic patterns via cross-immunity and antibody-dependent enhancement, but at the cost of greatly increased model and parameter complexity [49]. As our focus was on the effects of climatic forcing, we defer a full multi-strain extension to future work.

The sensitivity analyses of both the mosquito and complete models reveal that climate-dependent parameters, such as carrying capacity *C*(*t*), aquatic transition rate $\gamma_{m}(T(t))$ and bite rate *b*(*T*(*t*)), are the most influential in determining mosquito population dynamics and dengue incidence. Since the bite rate is multiplied by the infection rates between humans and mosquitoes, its effect, combined with the infection rates, significantly impacts the nonlinear terms of the complete model that govern the interaction between humans and mosquitoes.

Another important result from this study refers to the mosquito dynamics. The data suggest that mosquito abundance increased sharply from 2020 onwards. Initial versions of the model with constant or seasonal carrying capacity were not capable of representing this increasing abundance. Only a model with a linear trend of the carrying capacity correctly captured the observed pattern. Increasing carrying capacity indicates a relaxation of the density-dependence effects on mosquito population growth. Such a fact can occur if the number of breeding sites increases, thus reducing the competitive pressure in the aquatic stage. It is noteworthy that the 2020 - 2022 period corresponds to the first two years of the COVID-19 pandemic, during which significant changes occurred in human behavior and vector control efforts. Similar observations have been made in other locations [50]. We hypothesize that these changes may have contributed to alterations in mosquito abundance in Foz do Iguaçu.

We also investigated the temporal dynamics of dengue transmission and its relationship with climate variables, focusing on temperature, considering that it reflects more than other seasonal features. Through the complete model (1), instead of assuming the entomological parameter values corresponding to the average temperature as in [26], we consider their values for the daily temperature, according to some temperature-dependent functions [28,32]. While rainfall and humidity are known to influence mosquito dynamics, we restricted our environmental drivers to temperature due to the high noise and lack of significant correlations in the data at the municipal and weekly/bimonthly scale analyzed. Moreover, there is a predominance of temperature-based entomological parameterizations in the literature. Also, because the temperature-dependent mosquito parameters were adopted from literature, carried out in other regions, their transferability to the local vector population is uncertain. These simplifications are acknowledged as limitations of the model.

By analyzing the models, we compared the simulated human infection data with reported dengue cases from surveillance records (see Fig 8b), taking into account mosquito population dynamics and climate effects, as previously demonstrated by [51]. The confidence in the numerical results of the complete dengue model [26] is supported by the validation of the mosquito population dynamics model (2) against trapped mosquito data (see Fig 6), which allowed us to establish realistic entomological parameter values for use in the complete model.

The basic reproduction number, *R*_0_, indicates higher transmission in 2010 compared to 2020. This result underscores that neither the number of cases nor the incidence rate captures the information revealed by *R*_0_, which reflects the transmission dynamics at the beginning of an epidemic. Some hypotheses may explain this phenomenon. First, this pattern aligns with the introduction of new viral variants DENV-1 in 2010 and DENV-2 in 2020, as evidenced by dengue health surveillance data. A second hypothesis is that *R*_0_ estimates are more closely tied to the ecological characteristics of the vector associated with the circulation of non-specific serotypes [52]. Additionally, as discussed by [53], the timing of intervention implementation may influence the magnitude of the epidemic.

In this study, we also compared the estimation of *R*(*t*) using expressions that either include or do not include the temperature data. Despite the small difference in magnitude between the two estimates, our results indicate the expression incorporating temperature consistently produces higher values of *R*(*t*) compared to the one that does not. This implies the temperature-inclusive expression will more frequently exceed the transmission threshold (*R*(*t*) > 1), making it a more sensitive measure of *R*(*t*) for use in alert systems. These findings are consistent with the observations reported by [10].

As the disease progresses, several factors, such as control measures and changes in the susceptible population, can alter the scenario of disease dynamics so that it is less reasonable to consider its development in a constant environment with exponential growth of cases. Therefore, the time-dependent effective reproduction number, *R*(*t*), captures the temporal evolution of the reproduction number. Furthermore, ecological factors, urbanization, population mobility, deforestation, and insecticide resistance, together with climatic events and anomalies in recent decades, have created conditions for pathogens and arbovirus vectors to emerge in new areas or re-emerge in regions, even with powerful antimicrobial campaigns [22,54–56]. These heterogeneous characteristics also lead to different epidemic scenarios according to susceptibility and other factors [57].

## Conclusion

Our results call attention to the relevance of mosquito trap data in constructing a confidence time series of mosquito population dynamics to get a more accurate scenario of dengue dynamics. Both fitted models for Foz do Iguaçu display good fitting to data, showing a promising methodology for performing curve fitting with entomological parameters into a mosquito population model and also in a dengue transmission model. Particularly, this analysis may provide elements to help the description of dengue dynamics for other regions with sub-tropical climates where dengue infection is expanding. This framework can be used to plan interventions in pre-epidemic periods and generate timely alerts. The inclusion of the observable variable—trapped mosquitoes—in the model opens the possibility of developing triggers based on mosquito trapping thresholds. In Brazil, there is an effort to expand the use of trap-based entomological surveillance, which could provide more standardized data for this class of models. Finally, incorporating the trapped mosquitoes variable enables the generation of a more precise time series for the effective reproduction number, which monitors the epidemic character of the transmission process.

The results also highlight the role of climate variables, particularly temperature, directly affecting mosquitoes’ entomological characteristics or modifying human behavior, which, in turn, may impact the mosquito population. However, many other factors are relevant for the understanding of the complex dengue; in that direction, our results also emphasize that the climate alone does not explain either the increase of mosquitoes or the expansion of dengue.

Therefore, studies that consider the relationship between modeling and data, as well as the inclusion of additional elements in the model, are crucial. For instance, accounting for the co-circulation of dengue serotypes [49] and other diseases transmitted by the same vector, such as Zika and chikungunya [7], could lead to a more comprehensive description of dengue dynamics. This, in turn, would facilitate the design of more effective control strategies, including vaccination programs and vector control measures.
